# Prolonged Peripheral Hypoperfusion Promotes Neuroprotection in Ischemic Stroke

**DOI:** 10.7759/cureus.6105

**Published:** 2019-11-08

**Authors:** Caleb J Heiberger, Tej I Mehta, Stephanie Kazi, Gauravjot Sandhu, Divyajot Sandhu

**Affiliations:** 1 Radiology, University of South Dakota Sanford School of Medicine, Sioux Falls, USA; 2 Neurology, University of South Dakota Sanford School of Medicine, Sioux Falls, USA

**Keywords:** remote ischemic preconditioning, ischemic stroke, neuroprotection, peripheral vascular disease

## Abstract

Objectives

Sublethal, transient occlusion of peripheral vessels, called remote ischemic preconditioning (RIPC), induces a neuroprotective state against brain infarction. Recent studies suggest chronic hypoperfusion in patients with peripheral vascular disease (PVD) has analogous effects. We hypothesized a positive correlation between the severity of chronic hypoperfusion and the extent of neuroprotection. To determine if this correlation exists, we compared stroke volumes and clinical measures of modified ranking scale (mRS) and National Institute of Health Stroke Scale (NIHSS) between cases with and without PVD, subgrouping PVD cases by ankle-brachial-index (ABI) values.

Patients and methods

Cases of ischemic stroke with and without PVD were sampled retrospectively from a local institutional data base. Charts were manually reviewed for demographics (age, sex, ethnicity), comorbidities (diabetes, hypertension, hyperlipidemia, coronary artery disease, smoking, and stroke history), clinical measures (admission NIHSS, prior mRS, three-month mRS, and survival) and stroke volumes in each case. Those diagnosed with PVD and ABI indicating active disease were grouped as PVD cases; those not diagnosed with PVD or having ABI indicating absence of disease were used as controls. PVD cases were subgrouped by disease severity per ABI values: mild (ABI 0.8-0.9), moderate (ABI 0.5-0.9) and severe (ABI < 0.5). Data were analyzed in R using adjusted logarithmic-multivariate models. Adjusted cox proportional hazards models were used to estimate associations between survival and PVD.

Results

A total of 105 patients, 50 PVD cases and 55 controls, were collected. Mean age was 72.54 years, 51.4% were males and 48.6% females, and 94% were Caucasian. There were 17 mild, 22 moderate, and 11 severe cases of PVD. A higher incidence of comorbidities was present in PVD cases. The mean admission NIHSS was 4.44 and did not differ significantly between groups. Stroke volumes were significantly lower (p = .021) in PVD cases (4.39 ± 8.97 ml) compared to controls (19.33 ± 44.31 ml). There was also a significant difference (p = .04) between volumes of mild (3.86 ± 5.47 ml) and severe (0.63 ± 0.76 ml) PVD cases. There were significant differences (p = .012) in the incidence of good outcomes in moderate to severe PVD cases (100%) compared to controls (83.3%). There was no difference in survival between groups (p = .538).

Conclusion

Increasing degrees of hypoperfusion related to PVD have a potential neuroprotective effect in acute ischemic stroke quantified by lower stroke volumes and better clinical outcomes at three months as seen in other preclinical models of RIPC.

## Introduction

Ischemic stroke continues to be a leading cause of mortality and economic burden despite modern interventions. Ischemic tolerance (IT) is a cellular protective state that occurs following sub-lethal ischemic stimuli, a process known as ischemic preconditioning (IPC) [1]. Two distinct phases of IT are evident following IPC: 1) an acutely transient IT developing within minutes and lasting hours, mediated by post-translational modifications and 2) a delayed IT starting a day after phase 1 and offering up to a week of protection [2-4]. Models of IPC have been successfully applied to various organs, most notably the cardiac system. More recently, it has been applied to the brain with cerebral preconditioning (CPC).

Hyper/hypoxic, oxygen-glucose depriving, hypo/hyperthermic, and pharmacological IPC agents have CPC effects [5-11]. When used as an insult to primary neuronal cells, these agents trigger a messenger cascade resulting in cellular changes that produce IT. Potential mechanisms of CPC include resistance to glutamate excitotoxicity via an NMDA stimulation cascade involving nitric oxide, modulation of toll-like receptors suppressing the acute inflammatory response, and alterations in gene expression such as synthesis of heat shock proteins (e.g., HSP70) and transforming growth factors [12-20]. The list of “key players” involved in these pathways is beyond the scope of this introduction.

Aside from pharmaceutical agents, direct insult to neuronal cells has been the standard technique for inducing IPC. Further, pharmaceutical IPC requires use of drugs, i.e. flurane anesthetics, with associated deleterious effects. As such, the use of these CPC agents in clinical practice is limited. However, applying IPC to regions remote from cerebral neurons, called remote ischemic preconditioning (RIPC), avoids the risks seen in other techniques [21,22]. RIPC safety was studied in a population of critically ill patients, where no adverse effects were observed following its use [23]. Its mechanism, as studied in mouse models, is associated with reduced expression of IL-17 and IL-6 [24]. RIPC has demonstrated neuroprotective coverage during the interlude of the acute and delayed IT phases whereas other models have not [25].

Integrating RIPC in clinical practice remains challenging due to the erratic nature of cerebral infarction. However, successful clinical studies have been performed on smaller scales. Meng et al.’s study of RIPC applied after primary stroke in patients with intracranial stenosis found increased cerebral perfusion, decreased incidence of recurrent stroke, and faster recovery time after recurrent stroke [26]. In Gonzalez et al.’s study on a population of patients with subarachnoid hemorrhage, RIPC was linked with cerebral vasodilation and reduction in the lactate/pyruvate ratio [27]. Both studies used transient, intermittent episodes of sub-lethal peripheral occlusion to induce RIPC. Connolly et al. described a novel variant of RIPC achieved by chronic hypoperfusion as opposed to transient occlusion [28]. In their retrospective study, where peripheral vascular disease (PVD) was used as a mechanism for hypoperfusion, they illustrated improved clinical outcomes (lower admission National Institute of Health Stroke Scale (NIHSS) and three-month modified ranking scale (mRS)), smaller infarct volumes, and lower mortality rates in PVD afflicted patients.

Whether or not increasing degrees of hypoperfusion impacts RIPC in cases of ischemic stroke has yet to be studied. To address this question, we hypothesize that the neuroprotective effects, measured by clinical outcomes and stroke volume, in patients with PVD positively correlate with increasing degrees of hypoperfusion, indicated by lower Ankle Brachial Index (ABI) values.

## Materials and methods

Patient sampling

Approval to conduct the study and patient consent were approved by the institution’s review board. An institutional data base from a single center was retrospectively sampled from data spanning 10 years (2009 - 2018) to acquire a cohort of ischemic stroke patients. This center services over 500,000 people and is located in a major urban area. Two researchers independently reviewed charts, randomized by electronic medical record number, for data collection. Points of contention were resolved with a third party.

Inclusion criteria

Cases of ischemic stroke with or without a diagnosis of PVD were included in the study. Those without PVD were used as a control group. Presence of PVD was determined by both a diagnosis in the patient's chart and a corresponding ABI value indicating active disease. ABI values indicating PVD within a year of the stroke were considered proof of active disease unless a procedural intervention occurred in the interim. The most recent ABI available was chosen for the study.

Exclusion criteria

Those with a diagnosis of PVD but without a corresponding ABI within a year prior to the stroke or with an ABI indicating disease in the year prior but who had a procedural intervention after its dated occurrence were excluded from the study. Interventional treatment for stroke (TPA and endovascular therapies), absence of available DWI, presence of non-peripheral extracranial vascular disease (i.e., carotid and vertebral stenosis), and absence of NIHSS or mRS information were also grounds for exclusion. In cases of repeated stroke, the earliest stroke that met all other criteria was chosen. No duplicate patients were included.

Grouping

Those with ABI values indicative of PVD (ABI < 0.9) were labeled as cases and sub-grouped by severity: mild (ABI 0.8-0.9), moderate (ABI 0.5-0.8) and severe (ABI < 0.5). Those without a diagnosis of PVD and no ABI or having ABI values indicating absence of PVD (ABI > 0.9) were labeled as controls.

Sample characteristics

Demographics (age, sex, ethnicity) and comorbidities (diabetes mellitus - DM, hypertension - HTN, hyperlipidemia - HLD, smoking, coronary artery disease - CAD, and history of stroke) were recorded for all patients based off the admission note for their ischemic stroke.

Outcomes measures

The primary outcome measure was the difference in stroke volume. Secondary outcomes measures included change in mRS and admission NIHSS. Stroke volume, recorded in cubic centimeters, was calculated manually by reviewing each patient’s associated DWI. Volume was calculated by multiplying surface area of infarcted tissue on individual axial slices by total infarct thickness (number of slices x slice thickness + distance between slices). Surface area was measured using Centricity Universal Viewer’s line metric tool at the widest length and width of areas with apparent diffusion coefficient values corresponding to diffusion restriction from acute infarct. Measurements did not include T2 shine through. This technique was applied to all areas of infarct in multi-focal ischemic strokes. Prior mRS was assigned by reviewing the notes in the patient’s chart prior to stroke. Scores were assigned based upon patient functionality. Three-month mRS was reported as recorded in the patient’s chart. The change in mRS was equated to the three-month mRS minus the prior mRS. Outcomes were categorized as good (change in mRS ≤ 2) or bad (change in mRS ≥ 3). NIHSS and death were recorded as reported in the patient’s chart. Endpoint for outcomes was set for January 1st 2019.

Statistical analysis

Statistical analysis was performed using R. For univariate analyses, differences in continuous mean scores of demographic data and explanatory measures between the dichotomous summary measure of cases with PVD versus controls were examined using independent t tests for normally distributed data and the Mann-Whitney U test for skewed data. A multivariate model adjusted for confounding variables was generated to determine the significance and effect size of PVD on stroke volume and mRS scores. Fischer exact test was for categorical mRS comparison between cases of PVD and controls. Kaplan-Meir survival curves were generated to examine survival differences among cases with PVD and controls. Patients were censored if study participation was terminated for reasons other than death. Cox proportional hazard modeling was conducted to determine hazard ratios (HR) and adjusted hazard ratios for survival analysis.

## Results

Sampling and demographics

A total of 5,000 patients with diagnosis of ischemic stroke were identified from 2009 to 2018. During review, the first 60 cases of PVD and 60 cases without PVD meeting all criteria were included. Seven hundred and fifty-eight cases were reviewed in total. Two hundred and six cases were removed based upon exclusion criteria. The remaining 432 meeting criteria for the controls were not included in the study as the targeted sample size had been reached. Further review of the each cohort excluded 10 and five cases for the PVD and control cohorts respectively due to unidentifiable infarctions on DWI. In the final analysis, 105 cases were included: 50 with PVD and 55 without.

Age for the entire cohort ranged between 32 and 96, with a mean of 69.07 years. Cases with PVD were significantly older than controls (cases: 72.54 ± 10.98, controls: 65.91 ± 13.9, p = .008). Males comprised 51.4% of the study cohort and females 48.6%, with no significant differences between groups. Ethnicity did not differ significantly between groups. A 94% majority were from Caucasian descent, with American Indian and Hispanic comprising 12% and 1%, respectively. Comorbid conditions had significant (HTN, HLD, and AF) or near significant (DM and smoking) higher incidences in PVD cases, although history of stroke was not significantly different among groups. The mean admission NIHSS was 4.44 and did not differ significantly between groups. Mean prior mRS and three-month mRS were 0.26 and 1.39, respectively, with a difference of 1.14. The groups did not differ significantly in prior mRS values. The mean stroke volume of the study cohort was 12.22 ± 33.37 ml. Demographics and outcomes are displayed in Table 1 and Table 2.

**Table 1:** Characteristics and outcomes of cases and controls

Variable	Controls (n = 55)	Cases (n = 50)	p-diff
Diabetes, n (%)	20 (36.4)	27 (54.0)	.106
Hypertension, n (%)	39 (70.9)	48 (96.0)	.002
Hyperlipidemia, n (%)	25 (45.5)	45 (90.0)	
Atrial Fibrillation, n (%)	2 (3.6)	16 (32.0)	
Coronary Artery Disease, n (%)	12 (21.8)	29 (58.0)	
Smoking, n (%)	31 (56.4)	36 (72.0)	.144
Stroke History, n (%)	11 (20.0)	10 (20.0)	1
National Institute of Health Stroke Scale, mean (SD)	4.59 (4.44)	4.28 (4.47)	.720
Volume ml, mean (SD)	19.33 (44.31)	4.39 (8.97)	.021
Prior modified Ranking Scale, mean (SD)	0.25 (0.93)	0.26 (0.72)	.973
Three-month modified Ranking Scale, mean (SD)	1.53 (1.35)	1.24 (1.29)	.267
Change in modified Ranking Scale, mean (SD)	1.30 (1.11)	0.98 (1.15)	.157

**Table 2:** Characteristics and outcomes by Ankle Brachial Index

Variable	Control (n = 55)	Mild (n = 17)	Moderate (n = 22)	Severe (n = 11)	p-diff
Diabetes, n (%)	20 (36.4)	10 (58.8)	12 (54.5)	5 (45.5)	.286
Hypertension, n (%)	39 (70.9)	17 (100.0)	20 (90.9)	11 (100.0)	.006
Hyperlipidemia, n (%)	25 (45.5)	16 (94.1)	21 (95.5)	8 (72.7)	
Atrial Fibrillation, n (%)	2 (3.6)	6 (35.3)	8 (36.4)	2 (18.2)	
Coronary Artery Disease, n (%)	12 (21.8)	10 (58.8)	15 (68.2)	4 (36.4)	
Smoking, n (%)	31 (56.4)	13 (76.5)	14 (63.6)	9 (81.8)	.257
Stroke History, n (%)	11 (20.0)	5 (29.4)	4 (18.2)	1 (9.1)	.614
National Institute of Health Stroke Scale, mean (SD)	4.59 (4.44)	4.76 (5.40)	4.32 (4.69)	3.45 (1.97)	.873
Volume ml, mean (SD)	19.33 (44.31)	3.86 (5.47)	6.68 (12.30)	0.63 (0.76)	.001
Prior modified Ranking Scale, mean (SD)	0.25 (0.93)	0.12 (0.33)	0.50 (1.01)	0.00 (0.00)	.337
Three-month modified Ranking Scale, mean (SD)	1.53 (1.35)	1.47 (1.59)	1.23 (1.23)	0.91 (0.83)	.489
Change in modified Ranking Scale, mean (SD)	1.30 (1.11)	1.35 (1.62)	0.73 (0.77)	0.91 (0.83)	.173

Primary outcomes

Significant differences in the primary outcome were evident, with PVD cases having lower stroke volumes (cases: 4.39 ± 8.97 ml, controls: 19.33 ± 44.31 ml, p = .021). Multivariate analysis controlling for NIHSS and age found significant effects of NIHSS and PVD on stroke volume. The presence of PVD decreased mean stroke volume by 13.002 ml (p = .049) and a one-point increase in NIHSS resulted in a 1.552 ml increase in stroke volume (p = .032). Increasing degrees of PVD severity were associated with smaller stroke volumes: control (19.33 ± 44.31 ml), mild (3.86 ± 5.47 ml), moderate (6.68 ± 12.30), and severe (0.63 ± 0.76 ml). Significant differences in volume existed for severe (p < .001) and mild (p = .02) cases compared to controls and approached significance between moderate cases and controls (p = .062). Stroke volume in cases of severe PVD was significantly lower than either mild (p = .04) or moderate (p = .02) disease.

Secondary outcomes

Significant differences in secondary outcomes were evident when categorizing change in mRS by good (change in mRS ≤ 2) or bad (change in mRS ≥ 3). A higher but nonsignificant difference (p = .126) in incidence of good outcomes was seen in PVD cases (94%) over controls (83.3%). When excluding cases of mild PVD, there were significant differences (p = .012) in the incidence of good outcomes for PVD cases (100%) and controls (83.3%). Similarly, cases of moderate to severe PVD had significantly higher incidences of good outcomes compared to mild PVD (p = .022). Noncategorical analysis of both change in mRS (cases: 0.98 ± 1.11, controls: 1.30 ± 1.11, p = .157) and three-month mRS scores (cases: 1.24 ± 1.11, controls: 1.53 ± 1.35, p = .267) showed better outcomes in PVD cases compared to controls, although the difference was non-significant. Multivariate analysis for the change in mRS controlling for age, NIHSS, and gender found significant effects from NIHSS (1 point increase NIHSS = 0.138 point increase in change of mRS, p < .001) but not PVD (p = .30). Multivariate analysis for three-month mRS controlling for the same variable also showed significant effects from NIHSS (1 point increase NIHSS = 0.138 point increase in three-month mRS, <.001) but not PVD (p = .29). Unadjusted cox model for all-cause mortality indicated no difference in survival between cases and controls (HR 1.145, p = .538). In a multivariant analysis of survival controlling for age, gender, NIHSS, and comorbidities only NIHSS showed a significant impact on survival (p = .035).

## Discussion

The results indicate PVD is associated with lower stroke volumes and, in cases of severe to moderate disease, better clinical outcomes. Furthermore, severe PVD and moderate to severe PVD had lower stroke volumes and better clinical outcomes, respectively, compared to mild PVD. These findings are analogous to Connolly et al. and in addition indicate that the degree of hypoperfusion impacts outcomes. Contradictory to our findings, previous studies have demonstrated lower ABI values (more severe PVD) were associated with worse functional outcomes (mRS ≥ 3) in patients suffering from primary acute cerebral infarctions [29]. The higher incidence of comorbid conditions in patients with PVD places them at a disadvantage in regaining functional status. To control for this, we compared the change in mRS from prior to stroke to that at three-month follow-up between the groups. This consideration may account for the discrepancy between previous studies and our own. Noncategorical and multivariant analysis showed better clinical outcomes in cases of PVD, although the difference was nonsignificant. The loss of significance in these analyses is more reflective of previous literature and indicates the influence of extraneous variables on clinical outcomes. However, they may fail to indicate the true clinical outcome of the patient given the categorical nature of mRS scoring.

There was no difference in clinical scores at admission or rates of all-cause mortality through the study’s endpoint between cases of PVD and controls. The contrast in findings from Connolly et al. may be due to several factors. Connolly’s study paired PVD patients with a control by using a matching algorithm that considered demographics and comorbid conditions [28]. In our study, cases were not paired with matching controls and the higher incidence of premorbid conditions in the PVD cohort may have unfavorably impacted NIHSS scores. Whereas in mRS comparisons we controlled for this by considering mRS values prior to stroke, we did not do likewise for NIHSS.

Regarding mortality, Connolly’s argues that by excluding revascularized patients they may have limited cases of PVD to milder severities [28]. While our study had similar exclusions, reported ABIs indicative of persistent disease after vascular intervention were grounds for inclusion. ABIs of our cases indicate 66% had PVD severities in moderate to severe ranges. As reported in Kim et al.’s study, severe limb vascular insufficiency is related to worse outcomes in ischemic stroke populations [29]. It is possible the deleterious effects of severe limb ischemia outweigh the protective effects of preconditioning beyond the studied three-month functional assessment.

Our study is limited by its retrospective design. Randomization of cases by their medical record number was performed prior to chart review to reduce selection bias. Inadequate quality or absence of available imaging for stroke volumes precluded use of a number of cases, although this was similar for both groups. Efforts were made to assess the influence of extraneous variables by utilizing multivariant analysis to study outcomes. Although differences in age may have influenced stroke type and mortality, multivariate analysis controlling for age found significant effects of PVD on stroke volume. In data with large standard deviations, as seen in the stroke volume, Mann-Whitney U tests were used in lieu of independent t tests to adjust for skew.

Future works may further explore the point of critical hypoperfusion at which clinically significant outcomes following RIPC are evident. Trials comparing chronic hypoperfusion to transient sub-lethal total occlusion techniques would be valuable in developing protocols for clinical practice. Prospective, randomized clinical trials are being developed as an extension to this preliminary work.

## Conclusions

This retrospective study demonstrates that increasing degrees of chronic hypoperfusion related to PVD may promote better outcomes qualified by admission stroke volume and three-month mRS following acute ischemic infarction. This indicates that chronic hypoperfusion of the lower extremities may suffice as an agent for remote ischemic preconditioning and suggests a critical degree of hypoperfusion exists at which it occurs. It supports the existing literature that RIPC has a neuroprotective effect in acute ischemic stroke. To further explore the role of these findings in the clinical setting prospective, randomized, controlled trials should be developed.
